# Key Genes of the Immune System and Predisposition to Acquired Hemophilia A: Evidence from a Spanish Cohort of 49 Patients Using Next-Generation Sequencing

**DOI:** 10.3390/ijms242216372

**Published:** 2023-11-15

**Authors:** Jose Pardos-Gea, Laura Martin-Fernandez, Laia Closa, Ainara Ferrero, Cristina Marzo, Manuel Rubio-Rivas, Francesca Mitjavila, José Ramón González-Porras, José María Bastida, José Mateo, Marina Carrasco, Ángel Bernardo, Itziar Astigarraga, Reyes Aguinaco, Irene Corrales, Iris Garcia-Martínez, Francisco Vidal

**Affiliations:** 1 Systemic Autoimmune Diseases Unit, Department of Internal Medicine, Vall d’Hebron University Hospital, 08035 Barcelona, Spain; 2 Congenital Coagulopathies Laboratory, Blood and Tissue Bank, 08005 Barcelona, Spain; 3 Transfusional Medicine Group, Vall d’Hebron Research Institute, Autonomous University of Barcelona (VHIR-UAB), 08035 Barcelona, Spain; 4 Histocompatibility and Immunogenetics Laboratory, Blood and Tissue Bank, 08005 Barcelona, Spain; 5 Hematology Service, Arnau de Vilanova University Hospital, 25198 Lleida, Spain; 6 Department of Internal Medicine, Bellvitge University Hospital, Bellvitge Biomedical Research Institute (IDIBELL), University of Barcelona, L’Hospitalet de Llobregat, 08908 Barcelona, Spain; mrubio@bellvitgehospital.cat (M.R.-R.);; 7 Department of Hematology, Complejo Asistencial Universitario de Salamanca (CAUSA), Instituto de Investigación Biomedica de Salamanca (IBSAL), Facultad de Medicina, Universidad de Salamanca (USAL), 37007 Salamanca, Spain; 8 Thrombosis and Hemostasis Unit, Sant Pau Campus Salut Barcelona, 08025 Barcelona, Spain; 9 Hematology Service, Central University Hospital of Asturias, 33011 Oviedo, Spain; 10 Department of Pediatrics, Biobizkaia Health Research Institute, Hospital Universitario Cruces, University of the Basque Country UPV/EHU, 48903 Barakaldo, Spain; 11 Hematology Service, University Hospital Joan XXIII, 43002 Tarragona, Spain; 12 Centro de Investigación Biomédica en Red de Enfermedades Cardiovasculares (CIBERCV), Instituto Carlos III (ISCIII), 28029 Madrid, Spain

**Keywords:** rare bleeding disorders, Acquired hemophilia A, autoantibodies, next-generation sequencing, genetic predisposition to disease, immunogenetics

## Abstract

Acquired hemophilia A (AHA) is a rare bleeding disorder caused by the presence of autoantibodies against factor VIII (FVIII). As with other autoimmune diseases, its etiology is complex and its genetic basis is unknown. The aim of this study was to identify the immunogenetic background that predisposes individuals to AHA. HLA and KIR gene clusters, as well as *KLRK1*, were sequenced using next-generation sequencing in 49 AHA patients. Associations between candidate genes involved in innate and adaptive immune responses and AHA were addressed by comparing the alleles, genotypes, haplotypes, and gene frequencies in the AHA cohort with those in the donors’ samples or Spanish population cohort. Two genes of the HLA cluster, as well as rs1049174 in *KLRK1*, which tags the natural killer (NK) cytotoxic activity haplotype, were found to be linked to AHA. Specifically, A*03:01 (*p* = 0.024; odds ratio (OR) = 0.26[0.06–0.85]) and DRB1*13:03 (*p* = 6.8 × 10^3^, OR = 7.56[1.64–51.40]), as well as rs1049174 (*p* = 0.012), were significantly associated with AHA. In addition, two AHA patients were found to carry one copy each of the low-frequency allele DQB1*03:09 (*n*_allele_ = 2, 2.04%), which was completely absent in the donors. To the best of our knowledge, this is the first time that the involvement of these specific alleles in the predisposition to AHA has been proposed. Further molecular and functional studies will be needed to unravel their specific contributions. We believe our findings expand the current knowledge on the genetic factors involved in susceptibility to AHA, which will contribute to improving the diagnosis and prognosis of AHA patients.

## 1. Introduction

Acquired hemophilia A (AHA) is a bleeding disorder caused by the spontaneous formation of autoantibodies (inhibitors) against endogenous coagulation factor VIII (FVIII), which neutralize its coagulant activity (FVIII:C) [1,2]. This rare autoimmune disease has an annual incidence of 1.2–1.5 per million and its clinical presentation varies from life-threatening bleeds to mild or no bleeds [3,4]. Managing patients with AHA is extremely challenging, and an early and accurate diagnosis is crucial [5,6]. Approximately 50% of AHA cases are idiopathic; whereas in the remainder, a triggering disease or condition can be identified, including malignancies, autoimmune diseases, pregnancy, drug reactions, or dermatologic disorders [2].

The exact molecular mechanism that underlies the breakdown of tolerance to endogenous FVIII in AHA remains to be elucidated. However, there is evidence that this autoimmune response could be mediated by CD4+ T cell activation, and the contribution of these cells to anti-FVIII antibody response could be comparable to that observed in congenital hemophilia A (HA) [7,8,9]. As with other autoimmune pathologies, the etiology of AHA is thought to be complex, with both genetic and environmental risk factors being involved [10]. However, very little is known about the genetic background that confers susceptibility to developing this disease. A few studies have addressed the genetic basis of AHA, with most focusing on the FVIII-coding gene (*F8*) or immune response genes. Genetic variants in *F8* [11,12], in the cytotoxic T-lymphocyte-associated protein 4 gene (*CTLA4)* [10], and in genes encoding for the classical human leukocyte antigen (HLA) class II molecules [13] have been proposed to play a role in AHA. In particular, a higher frequency of the +49 A/G polymorphism in *CTLA4*, a negative regulator of T cell activation, was found in AHA patients [10]. This variant was suggested to cause a decrease in CTLA4 expression or function and lead to an upregulation of T cell responses. A significant association between AHA and HLA-DRB1*16 has also been reported, along with several nominal associations, among which are HLA-DRB1*15, HLA-DQB1*0602, and HLA-DQB1*0502 [13]. HLA molecules are crucial for antigen presentation to T cells, and thus, in the context of a breakdown in immunological tolerance to FVIII, it has been proposed that specific HLA alleles targeting particular FVIII self-antigens may predispose individuals to the development of AHA. In line with this evidence, common (c.3780C>G and c.8899G>A) and rare (c.6067G>A) variants in *F8* have been suggested to play a role in AHA [11,12]. These studies proposed that genetic variations compromising the primary structure of FVIII protein, in combination with specific HLA profiles, may lead to an aberrant antigen presentation and trigger inhibitor formation in AHA patients.

Both B and T cells are well-known key players in autoimmune diseases, and in recent years, evidence that natural killer (NK) cells may play a role has also been reported [14]. NK cells are cytotoxic lymphocytes that are involved in the innate immune response against pathogens and in the elimination of cancer cells. 

Their function is regulated by the combination of several inhibitory and activating cell-surface receptors and the balance of their signals [15]. Killer cell immunoglobulin-like receptors (KIRs) are one of the main NK cell inhibitory receptors encoded by the KIR locus (19q13.4). This polygenic complex is made up of 14 highly polymorphic genes that encode for both inhibitory and activating receptors and two pseudogenes. KIR receptors interact with HLA class I molecules [15]. In fact, these receptors or KIR/HLA genotypes have been associated with autoimmune diseases [14]. Similarly, the NKG2-D type II integral membrane protein (NKG2D), a C-type lectin-like receptor encoded by *KLRK1* (12p13.2), recognizes ligands expressed on stressed cells, such as MHC-class I polypeptide-related sequence A (MIC-A) and B (MIC-B) proteins. Engagement of this activating receptor along with its ligands triggers cell-mediated cytotoxicity and promotes the elimination of both transformed and infected cells [14,16]. Two different haplotypes of *KLRK1* have been linked to low (LNK) and high (HNK) NK cytotoxic activity [17]. Few studies have addressed the role of these key immune system players in autoimmune diseases, especially in AHA.

Traditionally, the genetic background underlying AHA has been studied using classical genetic tools and gene-based approaches, but these have proven insufficient for unraveling the contribution of genetics to AHA susceptibility. The recent advent of next-generation sequencing (NGS) has enabled the analysis of massive amounts of genomic data, which, in turn, has fostered a deep paradigm shift in the study of complex diseases. Accordingly, NGS may shed light on the potentially polygenic etiology of AHA.

In this context, the aim of our study was to examine several key genes implicated in the immune response using NGS. Specifically, the roles played by HLA and KIR gene clusters, as well as that of *KLRK1*, were evaluated in a Spanish cohort of 49 unrelated patients with AHA.

## 2. Results

### 2.1. Demographic and Clinical Profiles of the AHA Cohort

**Figure 1:**
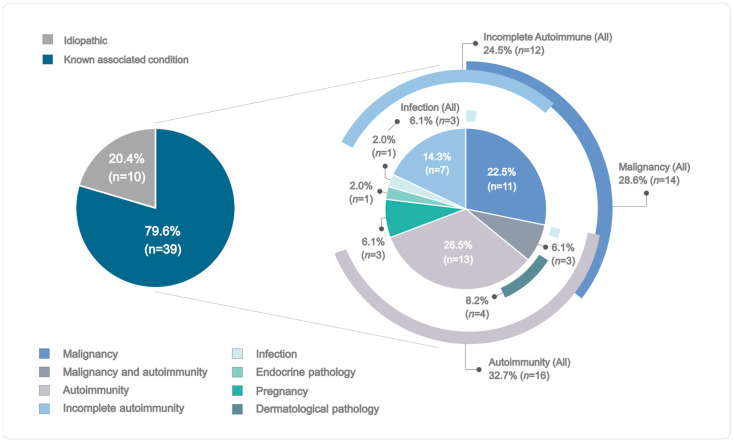
Conditions associated with AHA in 49 patients. Percentages in the pie chart are calculated for the entire sample. As several patients show different associated conditions concomitantly, total percentages considering all patients in a specific category are also displayed, which are noted as ‘All’ and represented by exterior circles that show the overlap with other categories. A more detailed description of the pathological conditions identified in these AHA patients is displayed in Supplementary Table S1).

The demographic and clinical information of the 49 patients with AHA are presented in Table 1. Briefly, 27 patients were males (55.11%) and 22 were females (44.89%), resulting in a male/female ratio of 1:0.81. The median age at diagnosis was 74 years (interquartile range [IQR] = 59–79.0). In most patients (*n* = 39, 79.59%), at least one AHA-triggering condition was identified, and only ten patients (20.41%) remained as idiopathic cases (Figure 1 and Supplementary Table S1). The median FVIII:C was 1 (IQR = 0.3–3.6) and the median FVIII inhibitor titer was 18 Bethesda units (BU)/mL (IQR = 5–62), with type II being the most prevalent type of inhibitor kinetics (*n* = 26, 53.06%). The median level of hemoglobin was 6.8 (IQR = 6–8.5) and the median activated partial thromboplastin time (aPTT) was 76 s (IQR = 62–90.8). Most patients had achieved complete remission by the time of data collection (*n* = 40, 81.63%). The median time in days to reach complete remission among patients that had achieved this status was 175 days (IQR = 84–266). The remaining patients had a partial remission (*n* = 7, 14.29%) or did not remit (*n* = 2, 4.08%). Overall mortality was 26.53% (*n* = 13) and AHA-specific mortality was 12.24% (*n* = 6). Of these cases, four patients (66.67%) died because of complications of immunosuppressive therapy (IST), such as infection, and two patients died as a consequence of both an infection after IST and fatal bleeding (33.33%). 

### 2.2. Hemostatic Treatment and Immunosuppressive Therapy

Information regarding hemostatic treatment was available for 38 out of the 49 AHA patients. Most of them required some form of hemostatic treatment (*n* = 31, 63.27%). The majority of patients needed a single hemostatic product to control hemorrhage (24, 77.42%), with the most common product being recombinant activated FVII (rFVIIa) (*n* = 18, 36.73%), followed by activated prothrombin complex concentrate (aPCC) (*n* = 5, 10.20%) and human FVIII (*n* = 1, 2.04%). A minority of patients needed combined therapy (*n* = 7, 22.58%) consisting of rFVIIa and human FVIII (*n* = 2, 4.08%), prothrombin complex concentrates (*n* = 1, 2.04%), aPCC (*n* = 3, 6.12%), or both human FVIII and aPCC (*n* = 1, 2.04%).

All AHA patients received immunosuppressive treatment (*n* = 49, 100%). The first IST regimen was effective in 32 patients (*n* = 32, 65.31%) who achieved complete remission and in 6 patients who achieved partial remission (*n* = 6, 12.24%). Ten patients needed a second (*n* = 9, 18.37%) or a third (*n* = 1, 2.04%) therapeutic regimen to be in complete remission, and one patient did not remit after the administration of the third therapeutic agent (*n* = 1, 2.04%). According to the last treatment received at the time of data collection, the most common form of IST was a combination of corticoids and cyclosporine (*n* = 14, 28.57%), followed by corticoids and tacrolimus (*n* = 10, 20.41%) or corticoids and cyclophosphamide (*n* = 9, 18.37%). Four patients received only cortisone (*n* = 4, 8.16%), another four were administered rituximab (*n* = 4, 8.16%), and three received a combination of corticoids and rituximab (*n* = 3, 6.12%). The remaining five patients were treated with cyclophosphamide (*n* = 2, 4.08%), plasmapheresis (*n* = 1, 2.04%), a combination of sirolimus and corticoids (*n* = 1, 2.04%), or a combination of bortezomib and cyclophosphamide (*n* = 1, 2.04%).

### 2.3. Genetic Analyses and Association Studies

#### 2.3.1. Classical HLA Class I and II Genes

For HLA class I genes, 21 different *HLA-A*, 26 *HLA-B*, and 17 *HLA-C* alleles were identified (Supplementary Table S2), with A*02:01 (*n*_allele_ = 24, 24.49%), B*44:03 (*n*_allele_ = 11, 11.22%), and C*04:01 (*n*_allele_ = 20, 20.41%) being the most common for each gene. For HLA class II genes, 23 different *HLA-DRB1*, 15 *HLA-DQB1*, and 16 *HLA-DPB1* alleles were detected (Supplementary Table S3), with the most common for each gene being DRB1*07:01 (*n*_allele_ = 16, 16.33%), DQB1*03:01 (*n*_allele_ = 21, 21.43%), and DPB1*04:01:01G (*n*_allele_ = 34, 34.69%).

Two alleles involving HLA class I or II genes were significantly associated with AHA (*p* < 0.05, Table 2). On the one hand, the frequency of A*03:01 was found to decrease in the AHA cohort compared to the donors, with carriers of this allele having 74% lower odds of developing this disease (odds ratio (OR) = 0.26; confidence interval (CI) = 0.06–0.85). On the other hand, the frequency of DRB1*13:03 was found to increase in the AHA cohort compared to the donors, with carriers of this allele being 7.6 times more likely to develop this disease. It is noteworthy that two AHA patients were found to carry one copy each of the DQB1*03:09 allele (*n*_allele_ = 2, 2.04%), which was completely absent in the donor sample. In addition, this allele was also absent in the Spanish population from the Allele Frequency Net Database (AFND) [18].

#### 2.3.2. Non-Classical HLA Class I and HLA Class I-like Genes

Eleven different alleles for *HLA-F* and fourteen alleles for *HLA-G* were identified (Supplementary Table S4) in the AHA patients. The most common alleles of each gene were F*01:01:01:08 (*n*_allele_ = 27, 27.55%) and G*01:01:01:01 (*n*_allele_ = 25, 25.51%). For *MICA* and *MICB*, 18 and 9 unique alleles were detected in the AHA cohort, respectively (Supplementary Table S5), with the most common alleles being MICA*004:01 (*n*_allele_ = 17, 17.35%) and MICB*005:02 (*n*_allele_ = 54, 55.10%).

None of the alleles involving non-classical HLA class I or HLA class I-like genes were associated with AHA.

#### 2.3.3. KIR Genes, Genotypes, and Haplotypes

KIR genes were successfully analyzed in 47 AHA patients (Supplementary Table S6). In the remaining two patients, amplification of the KIR cluster was incomplete and their data were, thus, excluded from the analyses. As expected, the frequencies of the KIR framework genes ranged from 97.87% to 100%. Inhibitory genes were more common among the AHA patients than activating genes, with the frequency of the latter ranging between 25.53 and 46.81%. The activating gene *KIR2DS4*, however, was present in all individuals. The frequencies of *KIR2DL1* and *KIR2DP1* were also 100%.

Thirteen different KIR genotypes were identified in our AHA cohort (Supplementary Table S7). The most frequent KIR genotype was ID.1 (*n* = 15, 31.91%). The AA haplotype was identified in 34.04% (*n* = 16) and the Bx haplotype was identified in the remaining 65.96% (*n* = 31) of the AHA cohort.

None of the associations of KIR genes, genotypes, and haplotypes with AHA reached the significance level.

#### 2.3.4. KLRK1 Gene

LNK and HNK dosage was inferred in all 49 AHA patients based on the rs1049174 genotype (Supplementary Table S8). The G allele, which tags the LNK haplotype, was the most frequent (*n* = 69, 70.41%). Most patients carried this variant in heterozygosis (CG; *n* = 29, 59.18%), whereas the homozygous GG genotype (LNK/LNK) was detected in 40.82% (*n* = 20). No patient had the CC wild-type genotype (HNK/HNK).

The statistical analysis revealed a significant global association between rs1049174 and AHA (Table 3). Specifically, the frequencies of the homozygous genotypes that tag either HNK/HNK or LNK/LNK haplotypes were lower in the AHA cohort compared to the Iberian population in Spain (IBS) from the 1000 Genomes Project, whereas the frequency of the heterozygous genotype was higher. Of these, the absence of the homozygous, wild-type genotype tagging the HNK haplotype and the excess of the heterozygous genotype found in the AHA patients were statistically significant (Table 3). 

## 3. Discussion

Our study is the most comprehensive performed to date that targets the immunogenetic basis of AHA. To the best of our knowledge, in addition to studying classical HLA I and II genes, this is the first work that addresses the contribution of non-classical HLA class I and HLA class I-like genes; KIR genes, genotypes, and haplotypes; and the *KLRK1* genotype to AHA. 

The demographic and clinical profiles of our AHA patients are consistent with studies in larger European and North American populations (Supplementary Table S9) [3,20,21,22,23,24,25,26]. The median age and male/female ratio of our patients were similar to those previously reported [20,22,23,25]. In line with previous studies, the most prevalent associated conditions were autoimmune diseases and malignancy [3,20,21,22,24]. Idiopathic cases, however, only accounted for 20.41% of the AHA sample, which implies that the proportion of patients in whom an associated condition could be established was almost twice what was expected based on previous reports (79.59%) (Figure 1, Supplementary Table S1). This can be explained by the extensive clinical and phenotypic characterization of our patients and, in particular, by the identification of a subgroup of patients being categorized as incomplete autoimmune. The median titer of FVIII inhibitors and its distribution by categories was also in accordance with earlier descriptions [4,23], as well as both the levels of hemoglobin and aPTT [20,23,24,25]. In contrast, the median FVIII:C level was within the lower range of previously reported values, and the proportion of patients with a severe phenotype (FVIII:C ≤ 1%) was notably higher [3,23,24]. The complete remission rate was higher in our AHA cohort, while overall and AHA-specific mortality rates were within the expected ranges [3,20,21,23]. Thus, the general characteristics of our cohort could be considered reliable and render the extrapolation of the results obtained in this genetic study to the overall AHA population plausible.

In our study, the classical HLA class I allele A*03:01 and the class II allele DRB1*13:03 were found to be associated with AHA. To the best of our knowledge, these are previously undescribed genetic factors associated with susceptibility to this disease. The association between HLA and autoimmunity has been known for decades, and the evidence is consistent with our findings [27,28]. Autoimmune diseases are characterized by immune disturbances that cause aberrant B and T cell reactivity to normal constituents of the host. However, the exact mechanisms that lead to the generation and activation of autoreactive cells have remained elusive for most of the autoimmune diseases. To date, several models have been proposed that focus on the fundamental role of HLA molecules in presenting a wide array of antigenic peptides to T cells, thus enabling the immune system to discern between self and non-self-antigens and maintaining tolerance [29]. These include low-affinity HLA–peptide–TCR binding, TCR-mediated stabilization of weak peptide–HLA interaction, and molecular mimicry.

In HA, the alloimmune response to therapeutic FVIII is suggested to result from the presentation of exogenous FVIII antigens to antigen-specific CD4+ T cells via HLA class II molecules, which, in turn, activates antigen-specific B cells [30]. Several studies have suggested that in HA, the failure of CD4+ T regulatory cell modulation of peripheral tolerance could also play a role, in addition to a loss of central tolerance to FVIII. In AHA, there is evidence that the autoimmune response against endogenous FVIII could also be mediated by CD4+ T cell activation and the contribution of these cells to anti-FVIII antibody response could be comparable to that observed in HA [7,8,9]. In this regard, *CTLA4* has been found to be associated with both forms of disease [10], as well as several HLA class II alleles, which have been reported to exert opposite effects between HA and AHA [31,32,33,34].

In this context, Uchanska-Ziegler et al. postulated that the product of HLA-A*03:01, which was found to be associated with AHA in our study, binds to a particular peptide set with high affinity during positive T cell selection in the thymus and leads to an abnormal T cell repertoire with autoreactivity that could predispose individuals to autoimmune disease [35]. HLA-A*03:01 is part of an ancestral haplotype (7.2) that has been implicated in susceptibility to multiple sclerosis and systemic lupus erythematosus and could play a protective role in type 1 diabetes [36]. Moreover, HLA-A*03:01 has been associated with a risk of multiple sclerosis in several studies, although there is debate on whether this effect might be due to HLA-A*03:01 linkage disequilibrium with the extended HLA haplotype DRB1*15:01~DQB1*06:02 [37]. Furthermore, Pavlova et al. detected a nominal association between HLA-A*03 and AHA in the same direction as we did in our study, which points to a protective role of this allele [13].

Regarding HLA-DRB1*13:03, which was also associated with AHA in our study, different publications have consistently reported its association with the risk of autoimmune diseases in spite of the low frequency that this allele generally presents in European populations, being close to 1% and rarely observed at frequencies greater than 3% worldwide [38]. In particular, HLA-DRB1*13:03 has been demonstrated to play a role in susceptibility to multiple sclerosis. In severe HA, HLA-DRB1*13:01 has been found to be associated with alloantibody development against exogenous FVIII in replacement therapy, which would suggest a common pathogenic mechanism involving specific HLA-DRB1*13 alleles for the development of either allo- or autoantibodies against FVIII [39]. Conversely, previous studies have proposed HLA-DRB1*13 as a universal protective factor against autoimmune diseases, possibly mediated by the presence of the DERAA sequence at positions 70–74 [38]. This apparent conflict with our results can be explained by the absence of the DERAA epitope in HLA-DRB1*13:03, which is contained, however, in HLA-DRB1*13:01, HLA-DRB1*13:02, and HLA-DRB1*13:04 [40]. Accordingly, HLA-DRB1*13:01 and HLA-DRB1*13:02 have been described as the main mediators of the protective effect in autoimmune diseases [38]. Therefore, HLA-DRB1*13:03 could be considered a new risk factor for AHA and, potentially, for other autoimmune diseases.

It is noteworthy to mention that two patients of our AHA cohort were found to carry one copy each of the allele HLA-DQB1*03:09 (2.04%), which was completely absent in the donor sample and in the Spanish population available in the AFND [18]. Previous studies support the involvement of specific HLA-DQB1*03 alleles and haplotypes in autoimmune diseases, including Sjögren’s syndrome, rheumatoid arthritis, and type 1 diabetes [27,41]. Several authors have shown that unstable HLA-DQ complexes can compromise peptide-binding and impair presentation of self-peptides, which results in the thymic escape of autoreactive T cells and a loss of central tolerance [29,42,43,44]. Furthermore, Praditpornsilpa et al. found that the haplotype DRB1*09-DQB1*03:09 is associated with antibody development against recombinant human erythropoietin in chronic kidney disease [45]. Unfortunately, the association of HLA-DQB1*03:09 with AHA could not be evaluated in the present study as the frequency of this allele in both the patient and the donor samples did not reach the threshold required for genetic analyses. Therefore, this finding especially warrants further follow-up in larger samples to confirm the potential involvement of HLA-DQB1*03:09 in AHA.

In contrast to previous studies performed by Pavlova et al. [13,31] in AHA patients, no associations involving DRB1*16, DQB1*05:02, DRB1*15, or DQB1*06:02 alleles with AHA were detected in our study. Differences in the typing methodology used, the level of allele resolution, and the genetic background between samples, among other factors, might have affected the associations being identified. In particular, DRB1*15 had been analyzed in previous studies at a low resolution, whereas we could differentiate at least two different alleles (DRB1*15:01 and DRB1*15:02) that showed distinct allelic distributions. The same applies to the association between DRB1*13:03 and AHA detected in our study, which had only been evaluated at a low resolution in earlier works, thus possibly diluting the specific effects exerted by this particular allele. In regard to DQB1*06:02, its frequency was two-fold higher in the AHA cohort compared to the reference sample, both in the study by Pavlova et al. and in our study. However, this difference did not reach a significant level in our analyses, probably due to our smaller sample size. Nevertheless, we uncovered a nominal association between HLA-A*03:01 and AHA. As noted above, this allele is in a strong linkage disequilibrium with the extended haplotype DRB1*15:01~DQB1*06:02 [37], and thus could be pointing to the same susceptibility signal. Regarding DRB1*16 and DQB1*0502, their role in AHA could not be evaluated in the present study because of their low frequency in both patients and donors.

In the present study, we also found a significant association between AHA and rs1049174 in *KLRK1*, which tags the NK cytotoxic activity haplotype. Compared to the IBS panel from the 1000 Genomes Project [19], our AHA cohort showed a lower frequency of the low-activity haplotype (LNK/LNK), an excess of the heterozygous combination (HNK/LNK), and a null frequency of the high-activity haplotype (HNK/HNK). Of these, the absence of the homozygous HNK haplotype and the excess of the heterozygous combination in the AHA patients were statistically significant. This distribution suggests a predominance of the NK low-activity phenotype in AHA. The activity of NK cells results from a balance between the genetic expression and functional diversity of activating and inhibitory signals from cell surface receptors and can either limit or exacerbate immune responses [15,46]. Recent studies have demonstrated that NK cells contribute to autoimmune diseases via one or more ways: by their involvement in the onset stage, maintenance of the autoimmune reaction, and/or by direct cell or tissue injuries [47]. Although most of the current evidence points to a disease-promoting role for these cells, several investigations have suggested that these cells can play both pathogenic and protective functions in autoimmune diseases, depending on the cell subset, microenvironment, and disease type and stage [47]. In line with our findings, different studies have provided evidence that the low activity of some NK cell subtypes, resulting from a reduction in either their number or function, could underlie autoimmune pathologies, such as systemic lupus erythematosus, Sjögren’s syndrome, multiple sclerosis, and type 1 diabetes [47]. Specifically, Benyamine et al. found that NKG2D expression and the percentage of NKG2D-expressing NK cells decreased in systemic sclerosis patients [48]. Taken together, these findings support a potential protective role for certain NK cell subsets in AHA and some autoimmune pathologies. In this model, dysfunction in the regulatory properties of NK cells could lead to a failure to control autoreactive T and B cell responses, thereby contributing to the pathogenesis of autoimmunity.

Further efforts are needed to elucidate the exact mechanisms by which the classical HLA class I allele A*03:01 and the HLA class II allele DRB1*13:03 may interplay with other candidate genetic or environmental factors to confer disease susceptibility. Future studies should aim at evaluating the structural compatibilities between the binding sites of AHA-associated HLA alleles and FVIII epitopes and how these complexes may drive or protect against loss of tolerance and immunopathology. Furthermore, it is essential to decipher the effective cytotoxic activity of NK cells according to their receptor repertoire, as well as the pathophysiological mechanisms by which these cells and the NKG2D receptor may be involved in AHA. 

In conclusion, our work represents the first holistic approach examining the role of pivotal immune system genes in AHA. Our findings improve the current knowledge of the genetic background involved in the susceptibility to this challenging disease. Increasing the sample size of our cohort of patients with AHA will allow a more in-depth assessment of the specific contribution of HLA-A*03:01, HLA-DRB1*13:01, and NKG2D to AHA and provide stronger evidence for the involvement of candidate, low-frequency genetic variants, such as HLA-DQB1*03:09. Understanding the complex molecular mechanisms that underlie these genetic associations and how these may compromise reciprocal crosstalk between immune cells and breakdown of self-tolerance will provide key biological insights into the pathogenesis of AHA. In the present era of personalized medicine, our findings expand the current knowledge, which will enable the early identification of at-risk patients and disease progression in the future.

## 4. Methods

### 4.1. Patients

This national, multicenter study involves eight Spanish hospitals: the Hospital Universitari Vall d’Hebron, the Complejo Asistencial Universitario de Salamanca, the Hospital Universitari de Bellvitge, the Hospital de la Santa Creu i Sant Pau, the Hospital Universitario Central de Asturias, the Hospital Universitari Arnau de Vilanova, the Hospital Universitario Cruces, and the Hospital Universitari Joan XXIII. Forty-nine unrelated Spanish patients diagnosed with AHA were included. Nineteen were recruited retrospectively and thirty were recruited prospectively from January 2018 to February 2020. This study was approved in 2017 by the Ethics Committee of the Hospital Universitari Vall d’Hebron. All participants provided informed consent.

The diagnostic criteria for AHA were (1) FVIII inhibitor titer > 0.60 BU/mL and (2) FVIII:C < 50%. Clinical and demographic information were recovered retrospectively for already-registered patients and prospectively for newly enrolled patients. At diagnosis, AHA patients were assessed with respect to sex, age, origin of the patient, related or triggering condition, bleeding cause (spontaneous, surgery, or trauma), FVIII inhibitor titer, FVIII:C levels, FVIII inhibitor kinetics (type I or II), hemoglobin level, aPTT, and bleeding severity (ISTH Bleeding Score) [49]. The hemostatic treatment and IST administered to these AHA patients were also recorded. Remission status and overall and specific mortality rates were evaluated at the time of data collection. Triggering conditions for AHA were classified as malignancies, autoimmune diseases (including autoimmune dermatologic conditions), infections, endocrine diseases, and pregnancy. An incomplete autoimmune entity was considered as a triggering condition for AHA if all the diagnostic criteria of an autoimmune disease were not met, but positivity for any of the serological markers (antinuclear antibodies, extractable nuclear antibodies, or rheumatoid factor, among others) was recorded. In the absence of a condition associated with AHA onset, the disease was considered idiopathic. The remission status was defined as partial in patients with stable FVIII:C levels > 50% and an absence of the inhibitor while still undergoing immunosuppressive therapy. Complete remission was defined according to the same criteria following the withdrawal of immunosuppressive therapy or in patients undergoing prednisone treatment at a dose of <10 mg/day. Specific mortality referred to deaths associated with bleeding or complications from immunosuppression therapy. Inhibitor kinetics were classified as type I in patients with a complete inhibition of FVIII:C, defined as a FVIII:C < 1% in any determination, or as type II when a residual activity level of FVIII:C > 1% was consistently measured. Continuous variables were expressed as medians and interquartile ranges, and categorical data were expressed as frequencies and proportions.

Some of the patients of the present study (*n* = 17) are also included in the Acquired Hemophilia A Spanish Registry (AHASR), the intent of which is to recruit and follow up with Spanish patients diagnosed with AHA. Recently, the initial scenario of AHA diagnosis and management in Spain, as well as an in-depth description and analysis of the clinical features that define the AHASR cohort and treatment efficacy, has been published and, thus, differs from the focus of our work [50].

### 4.2. Sample Collection and Phenotype Determinations

Coagulation studies were performed using peripheral venous citrated blood samples. aPTT was assayed using an automated system and SynthAsil reagent (HemosIL, Werfen, Barcelona, Spain). Plasma FVIII:C levels were assessed using a one-stage clotting assay or automated chromogenic assay using the Electrachrome FVIII kit (HemosIL, Werfen). Briefly, patients’ citrated plasma was added to FVIII-deficient plasma and incubated at 37 °C for 3 min. The reagent factor including IXa, X, thrombin, calcium, and phospholipids was added to the mix, which was incubated at 37° for 2 min. The chromogenic substrate was then hydrolyzed by activated factor Xa, liberating the chromophoric group, para-nitroaniline. The color was read photometrically. Inhibitor titer was quantified using the Bethesda method. Genomic DNA was extracted from EDTA-stabilized peripheral blood samples using a QIAsymphony DNA Mini Kit and a QIAsymphony SP instrument (Qiagen, Dusseldorf, Germany).

### 4.3. Candidate Region Amplification and Sequencing

Six classical HLA class I and II genes (*HLA-A*, *-B*, *-C*, *-DRB1*, *-DQB1*, and *-DPB1*), two non-classical HLA class I genes (*HLA-F* and *HLA-G*), two HLA class I-like genes (*MICA* and *MICB*), eight KIR inhibitory genes (*KIR2DL1*, *KIR2DL2*, *KIR2DL3*, *KIR2DL4*, *KIR2DL5*, *KIR3DL1*, *KIR3DL2*, and *KIR3DL3*), six activating genes (*KIR2DS1*, *KIR2DS2*, *KIR2DS3*, *KIR2DS4*, *KIR2DS5*, and *KIR3DS1*), and two pseudogenes (*KIR2DP1* and *KIR3DP1*) were amplified using multiplex long-range PCR with the SequalPrep long PCR kit with dNTPs (Thermo Fisher Scientific, Waltham, MA, USA) [51,52,53,54,55]. These regions were amplified using an in-house primer design. Four KIR genes (*KIR2DL4*, *KIR3DL2*, *KIR3DL3*, and *KIR3DP1*) were considered framework genes. Libraries were prepared using the NGSgo kit (GenDx, Utrecht, The Netherlands) according to the manufacturer’s instructions and sequenced using a MiSeq system using a 300-cycle (2 × 150) MiSeq reagent kit v2 (Illumina, San Diego, CA, USA).

HNK and LNK cytotoxicity haplotypes were tagged using the single-nucleotide variant rs1049174, located in the 3′UTR of *KLRK1* [56]. Based on the *KLRK1* genomic coding strand, the HNK haplotype was tagged by the wild-type allele C, and the LNK haplotype was tagged by the alternative allele G. The *KLRK1* 3′UTR was amplified in a single short PCR, with the indexes and adaptor sequences (single-direction 384 barcodes for Illumina, Fluidigm, South San Francisco, CA, USA) being simultaneously added in order to target the amplicons using the universal CS1 and CS2 sequence tags (Fluidigm). The pooled samples were purified using the MinElute gel extraction kit (Qiagen, Hilden, Germany) and sequenced using a MiSeq system with a 500 cycle (2 × 250) MiSeq reagent kit v2 (Illumina) [55].

### 4.4. Bioinformatics Analyses

Allele calling for classical HLA class I and II, non-classical HLA class I, and HLA class I-like genes was performed using the NGSengine software (v2.28.1; GenDx) based on the IMGT reference database (v3.51.0, respectively; GenDx), following the manufacturer’s specifications. HLA allele frequencies were collapsed into a 2-field assignment, except for *HLA-DPB1*, for which the G code was used, and *HLA-F* and *HLA-G*, for which a ≤4-field was designed.

The number and type of KIR genes were determined via in silico hybridization of gene-specific probes against the sequence reads, using an in-house bioinformatics pipeline [51]. KIR genotype ID and haplotype were classified using the AFND [18].

For rs1049174 in *KLRK1*, 3′UTR alignment and genotype calling were performed using the CLC Genomics Workbench software (v21; CLC Bio-Qiagen, Aarhus, DK) [55].

### 4.5. Reference Samples

The reference samples consisted of 190 unrelated, healthy blood and bone marrow donors who were residents in Catalonia and typed at the Banc de Sang i Teixits (BST, Barcelona). The AHA and the reference samples were sex matched. Classical HLA class I and class II data were available in a subset of 100 donors, whereas non-classical HLA class I, HLA class I-like, and KIR data were available in the remaining 90 donors [52,54]. For rs1049174 in *KLRK1*, the allele and genotype frequencies of the IBS panel obtained from the 1000 Genomes Project were used [19]. Additional data from the Spanish population available in the AFND were also used [18].

Genomic DNA was extracted from either EDTA-stabilized peripheral blood samples or stabilized saliva samples using the QIAsymphony DNA Mini Kit or the QiaSymphony DNA Midi Kit on a QIAsymphony SP instrument (Qiagen, Dusseldorf, Germany). For all genetic analyses of each of the donor samples, the NGS-based protocols and bioinformatics pipelines were the same as for the AHA patients.

### 4.6. Statistical Analyses

To identify both risk and protector genetic factors for AHA, a minimum frequency of 0.05 in the AHA or reference samples was considered for the statistical analyses. Association with AHA was examined by comparing the alleles, genotypes, haplotypes, and gene frequencies in the AHA cohort with those in the corresponding reference sample. Statistical significance was assessed using Fisher’s exact test implemented in the *exact2x2* package [57] for pairwise comparisons and in the *stats* package for contrasts involving more than two groups. All statistical analyses were performed in the R environment (version 3.6.1, https://www.r-project.org/, accessed on 2 June 2023). The significance threshold was set at *p* = 0.05.

## Figures and Tables

**Table 1:** Demographic and clinical features of 49 patients with AHA. AHA: acquired hemophilia A; *n*: number of patients; IQR: interquartile range; BU: Bethesda units; FVIII: factor VIII; aPTT: activated partial thromboplastin time. * Data available for 47 patients with AHA. ^†^ Data available for 31 patients with AHA. ^‡^ Includes fatal bleeding and complications from immunosuppressive or hemostatic therapy. ^§^ Data available for 38 patients with AHA.

**A. General Characteristics of the AHA Cohort**		
Sex, *n* (%)	Male	27 (55.1)
	Female	22 (44.8)
	Male: female ratio	1:0.81
Age at diagnosis (years)	Median (IQR)	74 (59–79)
Cause of bleeding at onset, *n* (%) *	Spontaneous	41 (83.7)
	Surgery	2 (4.1)
	Trauma	3 (6.1)
	Peripartum	1 (2.0)
ISTH bleeding score *	Median (IQR)	8 (6–11)
	Males	8.8
	Females	7.9
Remission status at data capture, *n* (%)	Complete	42 (85.7)
	Partial	6 (12.2)
	No remission	1 (2.0)
Time to complete remission (days) ^†^	Median (IQR)	175 (84–266)
Death at data collection	Overall mortality, *n* (%)	13 (26.5)
	AHA specific, *n* (%) ^‡^	6 (12.2)
**B. Laboratory Parameters of the AHA Cohort at Diagnosis**		
Hemoglobin (g/dL)	Median (IQR)	6.8 (6.0–8.5)
aPTT (s) ^§^	Median (IQR)	76 (62–90.8)
FVIII:C (%)	Median (IQR)	1 (0.3–3.6)
FVIII level category, *n* (%)	Severe (≤1%)	28 (57.1)
	Moderate (>1 to ≤5%)	12 (24.5)
	Mild (>6 to ≤50%)	9 (18.4)
Inhibitor titer (BU/mL)	Median (IQR)	18 (5–62)
Inhibitor titer category, *n* (%)	0 to ≤10 BU/mL	19 (38.8)
	>10 to ≤100 BU/mL	22 (44.9)
	>100 to ≤1000 BU/mL	8 (16.3)
Inhibitor kinetics type, *n* (%)	Type I	23 (46.9)
	Type II	26 (53.1)

**Table 2:** Significant associations for classical HLA class I and II alleles in 49 patients with AHA. AHA: acquired hemophilia A; Freq: frequency; CI: confidence interval. * Reference sample consisting of 100 unrelated and healthy blood and bone marrow donors, who are residents in Catalonia.

Allele	Allele Count in AHA Cohort	Allele Freq (%) in AHA Cohort	Allele Count in Reference Sample *	Allele Freq (%) in Reference Sample *	Fisher *p*-Value	Odds Ratio (95% CI)
A*03:01	3	3.06	22	11.00	0.024	0.26 (0.06–0.85)
DRB1*13:03	7	7.14	2	1.00	6.82 × 10^3^	7.56 (1.64–51.40)

**Table 3:** Associations obtained from the analysis of the distribution of rs1049174 genotypes in *KLRK1* in 49 patients with AHA. AHA: acquired hemophilia A; Freq: frequency. * Reference sample consisting of 107 individuals of the Iberian population in Spain (IBS) from the 1000 Genomes Project [19].

Rs1049174 Genotype	Haplotype	Count in AHA Cohort	Freq (%) in AHA Cohort	Count in IBS *	Freq (%) in IBS *	Fisher *p*-Value	Odds Ratio (95% CI)	Fisher *p*-Value for Global Association
CC	HNK/HNK	0	0.00	10	9.35	0.031	0 (0–0.89)	0.012
CG	HNK/LNK	29	59.18	42	39.25	0.024	2.23 (1.09–4.58)	
GG	LNK/LNK	20	40.82	55	51.40	0.232	0.65 (0.32–1.34)	

## Data Availability

The datasets presented in this article are not readily available because all relevant data are contained within the article. Due to patient confidentiality and participant privacy, no additional data can be made publicly available. Therefore, the generated datasets will not be made available upon request.

## Supplementary Materials

The following supporting information can be downloaded at https://www.mdpi.com/article/10.3390/ijms242216372/s1. References [3,20,21,22,23,24,25,26,50,58,59] are cited in the supplementary materials.
